# Proof-of-Concept Nanoparticle-Based Biosensor for Detecting the African Swine Fever Virus Across Multiple Genotypes Using In Silico and In Vitro Approaches

**DOI:** 10.1021/acsomega.5c05375

**Published:** 2025-10-22

**Authors:** Chelsie Boodoo, Evangelyn C. Alocilja

**Affiliations:** Department of Biosystems and Agricultural Engineering, 3078 Michigan State University, East Lansing, 48824 Michigan, United States

## Abstract

African swine fever virus (ASFV) is a viral hemorrhagic disease with high lethality in domestic and wild swine, posing a critical threat to global food security and livestock economies. Rapid and accurate detection of ASFV is crucial for effective containment of outbreaks. This study evaluated a gold nanoparticle-based biosensor for the detection of ASFV by targeting the p72 gene using eight oligonucleotide probes. The objective was to identify optimal probes with high sensitivity and specificity, and broad genotypic coverage. Clustal Omega was used to perform multiple sequence alignments between each probe and diverse ASFV genomes. Percentage identity matrices were generated and visualized through heatmaps to assess hybridization strength across genotypes. The biosensor was then tested with synthetic ASFV DNA at a 5 min reaction time, using spectrophotometric analysis to evaluate detection. Sensitivity was measured through serial dilutions of target DNA, and specificity was confirmed using nontarget bacterial DNA. Probes 2 (40 bp, 50.0% GC content) and 5 (60 bp, 54.2% GC content) demonstrated the strongest overall performance, achieving detection of 550 copies with no cross-reactivity and strong binding across multiple ASFV genotypes. Statistical analysis using Spearman’s rank correlation demonstrated that GC content was significantly associated with sensitivity (ρ = −0.80, *p* = 0.016), while probe length, secondary structure stability, and binding advantage showed no significant relationships. This study underscored the importance of integrating genomic alignment tools with experimental biosensor validation to enhance probe design.

## Graphical abstract

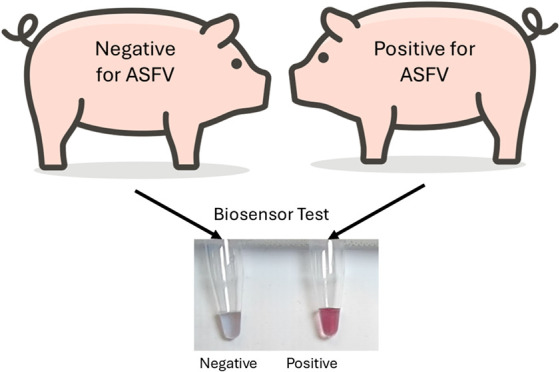

## Introduction

African swine fever (ASF) is a highly contagious and lethal viral disease affecting domestic and wild swine. The disease is caused by the African swine fever virus (ASFV), a large 170–194 kilobase pair (kbp) linear double-stranded DNA virus belonging to the *Asfarviridae* family.^1^ ASFV has led to devastating outbreaks worldwide, with mortality rates reaching nearly 100% in susceptible pig populations. Since its first recorded case in Kenya in 1921, ASF has spread beyond its endemic regions in sub-Saharan Africa to Europe, Asia, and the Americas, posing a significant threat to global food security and the pork industry.^2,3^ The continued expansion of ASFV highlights the urgent need for effective diagnostic tools to enable early detection and containment of the virus.^4^

One of the unique characteristics of ASFV is its ability to survive for long periods in the environment, making it particularly challenging to eradicate once introduced into a region.^5,6^ Clinically, ASFV manifestations in infected pigs include high fever, loss of appetite, hemorrhages in the skin and internal organs, and ultimately, death.^7^

The economic consequences of ASF outbreaks are severe. Countries experiencing outbreaks face major trade restrictions, significant livestock losses, and disruptions in pork production. The United States, which has one of the world’s largest pork industries, is particularly vulnerable to ASF introduction. A widespread outbreak is projected to result in economic losses exceeding 50 billion USD over the next decade, with approximately 140,000 job losses and long-term disruptions to the agricultural sector.^8,9^ Despite stringent biosecurity measures, ASFV remains difficult to control due to its ability to persist in the environment, its transmission through contaminated feed and fomites, and the absence of a widely available vaccine.^5,10^ While a live attenuated vaccine was recently approved for use in Vietnam, its global applicability remains uncertain, necessitating alternative strategies for ASFV detection and containment.^11^

Rapid and reliable diagnostic methods are critical for controlling ASF outbreaks. Current gold-standard methods, such as polymerase chain reaction (PCR) and enzyme-linked immunosorbent assays (ELISA), provide high sensitivity and specificity but require laboratory infrastructure, trained personnel, and extended processing times.^12^ These requirements limit their feasibility for field-based surveillance, particularly in resource-limited settings. Biosensors offer a promising alternative, enabling rapid, portable, and cost-effective detection of ASFV. Gold nanoparticle (GNP)-based biosensors leverage the unique optical properties of GNPs to facilitate visual detection of target DNA sequences with high sensitivity.^13^ By functionalizing GNPs with oligonucleotide probes complementary to ASFV genomic regions, these biosensors provide a colorimetric detection platform that is simple, robust, and potential to be field-deployable.^14,15^

GNP-based biosensors have gained significant attention in recent years due to their adaptability in detecting a variety of biological targets.^16^ Leveraging GNPs in this ASFV biosensor underscored the platform’s broad applicability across different pathogen detection needs, as the unique properties of GNPs enabled sensitive and specific molecular recognition. GNPs are widely used in biomedical applications, including sensing,^13,17^ drug delivery,^18^ and cellular imaging.^19,20^ They offer several advantages such as low-cost synthesis,^21,22^ high chemical and physical stability, nontoxicity, ease of surface functionalization with organic and biological molecules.^23^ These characteristics make GNPs ideal for use in biosensors. One of the most important properties of GNPs is their optical behavior driven by localized surface plasmon resonance (LSPR).^13,24^

LSPR occurs when light interacts at the surface of the GNPs, exciting the surface electromagnetic waves. This resonance amplifies light absorption at specific wavelengths, giving GNPs their distinctive optical properties.^25,26^ The size of the GNPs plays a crucial role in this phenomenon, as it influences the intensity and frequency of the absorption band, which directly affects the LSPR behavior.^13,27–29^ Larger particles scatter and absorb more photons, which modifies the color and optical response of the nanoparticles.

GNPs use LSPR-based light absorption and scattering. By modifying the surface of GNPs with specific biological molecules, these nanoparticles can be tailored to detect target molecules with high sensitivity and specificity. This makes GNP-based biosensors powerful tools for applications like pathogen detection, including the detection of the ASFV in this study.

A key challenge in developing molecular diagnostics for ASFV is the genotypic diversity of the virus. Historically, ASFV has been classified into 24 genotypes based on the p72 gene, which encodes the major capsid protein and is among the most conserved regions of the ASFV genome.^30^ This genotypic variation raises concerns about whether existing molecular assays can reliably detect all strains. Therefore, probe-based diagnostics must be evaluated for their ability to hybridize across these diverse genotypes to ensure broad applicability and high diagnostic coverage.

This study aimed to design a GNP-based biosensor for ASFV detection by evaluating oligonucleotide probes targeting the p72 gene across established ASFV genotypes. Eight oligonucleotide probes were assessed for their sensitivity and specificity using synthetic ASFV DNA. Multiple sequence alignments were performed using Clustal Omega^31^ to generate percentage identity matrices, which were visualized through heatmaps to determine probe–genotype hybridization efficiency. The goal was to identify probes that provide robust and accurate detection of ASFV across genetically diverse strains. In addition, statistical analysis was applied to test whether probe features such as GC content, length, and predicted secondary structure were associated with biosensor sensitivity. This study’s findings provide critical insights into the development of rapid, field-deployable biosensors and contribute to ongoing efforts in ASFV surveillance and outbreak prevention.

## Materials and Methods

### Genotypic Coverage and Hybridization Analysis

As the first step in probe evaluation, multiple sequence alignments were conducted using Clustal Omega to assess the hybridization efficiency of the eight candidate probes across ASFV genotypes. Clustal Omega uses a progressive alignment strategy enhanced with Hidden Markov Models (HMMs) profile–profile alignments. A percentage identity matrix was generated from these alignments and analyzed using Python. Only genotypes with at least partial hybridization to one or more probes were retained, while accession numbers with NaN values for all probes were omitted to focus the analysis on genotypically relevant sequences.

Bar plots were generated to display the mean percentage identity for each probe generated from Clustal Omega, offering a direct comparison of binding efficiency across ASFV genotypes. Error bars representing standard deviations were included to capture variability in hybridization performance. Heatmaps were employed to visualize genotypic coverage and probe binding efficiency across ASFV genotype classifications. The intensity of color shading in these heatmaps reflected percentage identity, with darker shades indicating stronger probe hybridization. This method enabled a comparative analysis of probe performance, highlighting cases where probes exhibited strong binding to multiple genotypes or limited hybridization to a narrow subset.

### Synthetic DNA and Probe Preparation

Synthetic DNA corresponding to the p72 gene of ASFV was obtained in lyophilized form from BioGx. Each lyophilized bead contained 100,000 copies of ASFV p72 DNA. The DNA was reconstituted in nuclease-free water and serially diluted to obtain working concentrations ranging from 4400 copies to the lower detection limit.

Eight oligonucleotide probes targeting conserved regions of the ASFV p72 gene were selected. Probes ranged from 28 to 80 base pairs in length and were provided by a collaborator. Each probe was diluted to a working concentration of 25 μM and stored at −20 °C to preserve stability. The GNP biosensor assay was optimized for a 5 min detection window to ensure rapid results.

### Gold Nanoparticle (GNP) Synthesis and Functionalization

GNPs were synthesized following the dextrin reduction method as previously described by Yrad et al.^32^ The synthesis of dextrin-coated GNPs involved the reaction of 5 mL of 2 mM gold­(III) chloride trihydrate (HAuCl_4_·3H_2_O) in 39.5 mL of sterile water, followed by the addition of 0.5 mL of 10% sodium carbonate (Na_2_CO_3_) as a reducing agent. The resulting dextrin-coated GNPs were functionalized with 25 μM 11-Mercaptoundecanoic acid (MUDA) and resuspended in 500 μL borate buffer for conjugation with the oligonucleotide probes.

### Experimental Design for Sensitivity and Specificity Evaluation

To evaluate probe performance, the GNP biosensor assay was designed to assess both sensitivity and specificity. Sensitivity was assessed by using serial dilutions of ASFV p72 synthetic DNA, ranging from 4400 copies to the lowest detectable concentration. Specificity was determined using nontarget DNA samples: *Escherichia coli* O157, *Salmonella enterica* serovar Enteritidis, and *Staphylococcus aureus* were prepared at a concentration of 20 ng/μL (approximately 4400 ASFV p72 DNA-equivalent copies based on molecular weight and base pair calculations). Nuclease-free water served as a negative control.

Three high-abundance barnyard bacteria were selected, *S. aureus* (Gram-positive), *E. coli* and *S. Enteritidis* (Gram-negative), as nontargets because they are prevalent in swine production environments, including oral fluids, feces, pen-surface dust, and feed dust. Air and surface surveys of pig houses repeatedly report *Staphylococcus spp.* among dominant or clinically relevant airborne bacteria and feed, indicating regular oral and environmental exposure in swine facilities.^33–35^ Farm monitoring similarly detects *S. aureus, E. coli* and *S. Enteritidis* in aerosols and settled dust across livestock operations, including swine.^36–38^ *Staphylococci* are commonly detected in pig saliva,^39^ while *E. coli* and *Salmonella* frequently contaminates swine feces.^40,41^ These organisms therefore represent realistic, high-burden interferents for field use. Beyond mere presence, bacterial cell-wall components and matrix polysaccharides can perturb GNP colorimetry. Lipopolysaccharide (LPS) is itself a classic inducer of GNP aggregation in endotoxin color tests, which demonstrates the potential for spurious red-to-blue shifts in contaminated samples.^42^ Using abundant Gram-positive and Gram-negative bacteria as stressors thus provides a stringent check against nonspecific aggregation, biofouling, and matrix-triggered false positives.

Note that RNA swine viruses such as classical swine fever virus (CSFV), porcine reproductive and respiratory syndrome virus (PRRSV), and porcine epidemic diarrhea virus (PEDV) are biologically relevant coinfections but present minimal sequence homology to the DNA probe targets in this study; in a hybridization-only assay that does not reverse transcribe RNA, these viruses are unlikely to challenge nucleic-acid specificity. In contrast, barnyard bacteria directly challenge the chemical robustness of the GNP readout in the very matrices where the test would be deployed, which is the central risk for colorimetric field biosensors. Despite CSFV, PRRSV (types 1 and 2), and PEDV being RNA viruses, probe cross-reactivity was assessed using NCBI BLASTN. Sequence homology searches were limited to target taxa using NCBI Taxonomy identifiers (CSFV = 11096, PRRSV-1 = 1965066, PRRSV-2 = 1965067, PEDV = 28295). BLAST analyses were conducted against the nucleotide (nt) database with the following parameters: word size of 7, both strands searched, low-complexity filter disabled, and an E-value threshold of 1000. Additionally each probe was screened against CSFV (accession: NC_002657.1), PRRSV (accession: PV948008.1, NC_038291.1), and PEDV (accession: OF367717.1), and computed an alignment-free 8-mer containment; both analyses returned no matches.

### GNP Biosensor Assay and Spectrophotometric Detection

The independent variables in this study included the type of DNA sample, which could be target ASFV p72 DNA, nontarget bacterial DNA, or a negative control (nuclease-free water), as well as the oligonucleotide probe used in each reaction, which varied in length and sequence. The dependent variables were the biosensor’s colorimetric response, where a red color indicated positive detection and a gray-blue color suggested nontarget DNA or absence of ASFV DNA, and the wavelength shift from 520 nm, which indicated probe-target mismatch and GNP aggregation. To ensure reproducibility and accuracy, controlled parameters included reaction volumes of 10 μL of template DNA, 5 μL of GNPs, and 5 μL of oligonucleotide probe. The thermocycler conditions were standardized with denaturation at 95 °C for 5 min, annealing at 55 °C for 10 min, and cooling at 25 °C. Acid-induced aggregation with 0.1 M HCl was also performed to facilitate detection.

Hybridization of ASFV DNA with a complementary probe prevented aggregation, maintaining a red color, whereas nontarget DNA or absence of target DNA led to GNP aggregation, resulting in a visible color shift from red to gray-blue, which is demonstrated in Figure 1. Absorbance spectrum was recorded using a NanoDrop One-C spectrophotometer, where the presence of ASFV DNA was indicated by a peak at 520 nm, while shifts in peak wavelength signified probe-target mismatch and GNP aggregation.

**Figure 1:**
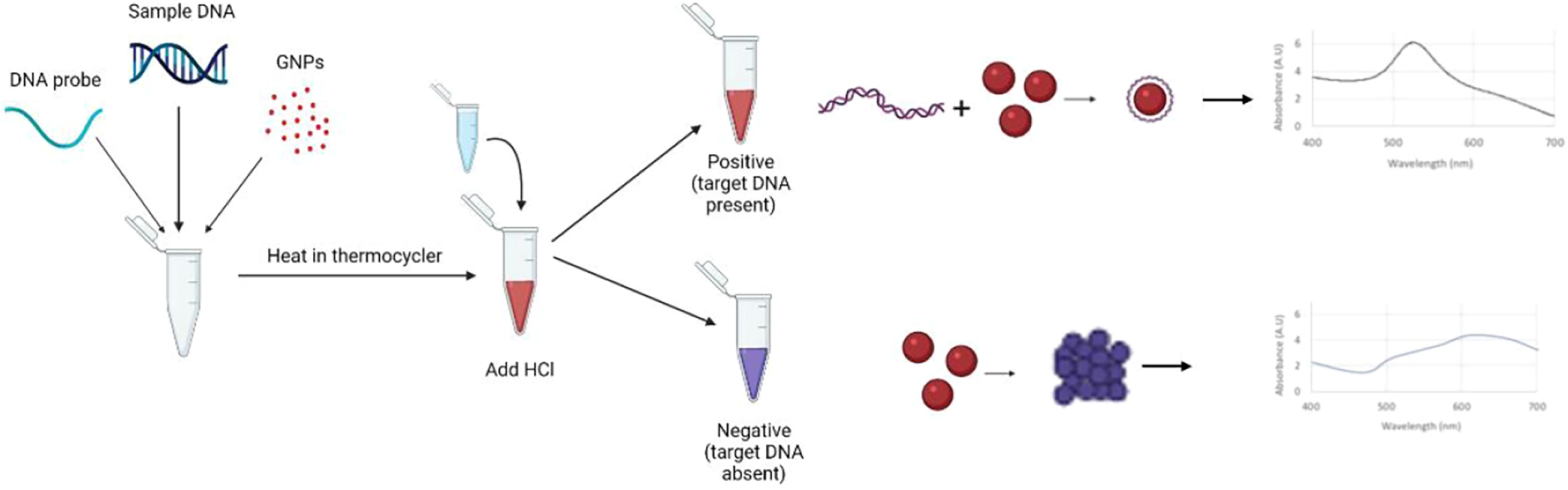
Protocol for ASFV biosensor. The protocol for creating the GNP biosensor and interpreting its results.

### Statistical Analysis, Probe Features and Correlation Analysis

Two-way ANOVA with Tukey’s multiple comparisons test was used to assess statistically significant differences in probe specificity and sensitivity (*p* < 0.05). Exact p-values, mean differences, and degrees of freedom for the two-way ANOVA comparisons are provided in Table S1. Spearman’s rank correlation was applied to evaluate associations between probe features and sensitivity (LOD, copies/μL). Independent variables included probe length, GC content, and secondary structure stability metrics (hairpin Δ*G*, self-dimer Δ*G*, and heterodimer Δ*G*).

Secondary structure stability was predicted using IDT OligoAnalyzer and NUPACK/UNAFold under assay conditions (55 °C, assay ionic strength). For each probe, hybridization free energy (Δ*G* target) was calculated to represent duplex stability. The minimum self-folding free energy (Δ*G* self,min) was defined as the most stable hairpin or self-dimer. Binding advantage (ΔΔ*G* adv = Δ*G* target – Δ*G* self,min) represented the favorability of target binding over self-structure formation. Duplex *T*_m_ was calculated to confirm that probe melting exceeded assay temperature. All correlation analyses and data visualization were performed in Python. Full scripts for data preprocessing, correlation, and figure generation are provided in Appendix B.

## Results

### Initial Probe Validation Using Clustal Omega

To confirm the potential hybridization efficiency of the eight designed oligonucleotide probes, Clustal Omega was first used to perform multiple sequence alignments between each probe and representative ASFV genomes spanning traditional genotypes. This computational step served as the foundation for all subsequent analyses, allowing for rapid, in silico prediction of probe-target interactions. Clustal Omega generated percentage identity matrices that revealed hybridization potential, and the results of this preliminary alignment confirmed that all probes demonstrated at least partial complementarity to one or more ASFV genotypes.

These data were visualized in Figure 2, which presents the average percentage identity across all ASFV genotypes for each probe, with error bars representing standard deviation. Probes 5 and 6 exhibited the highest mean identity values (∼61%), followed by Probes 2 and 3 (∼56 and 50%, respectively). In contrast, Probes 1, 4, 7, and 8 averaged lower (∼46–51%), indicating comparatively weaker binding potential. These results suggested Probes 2, 5, and 6 had comparatively stronger binding.

**Figure 2:**
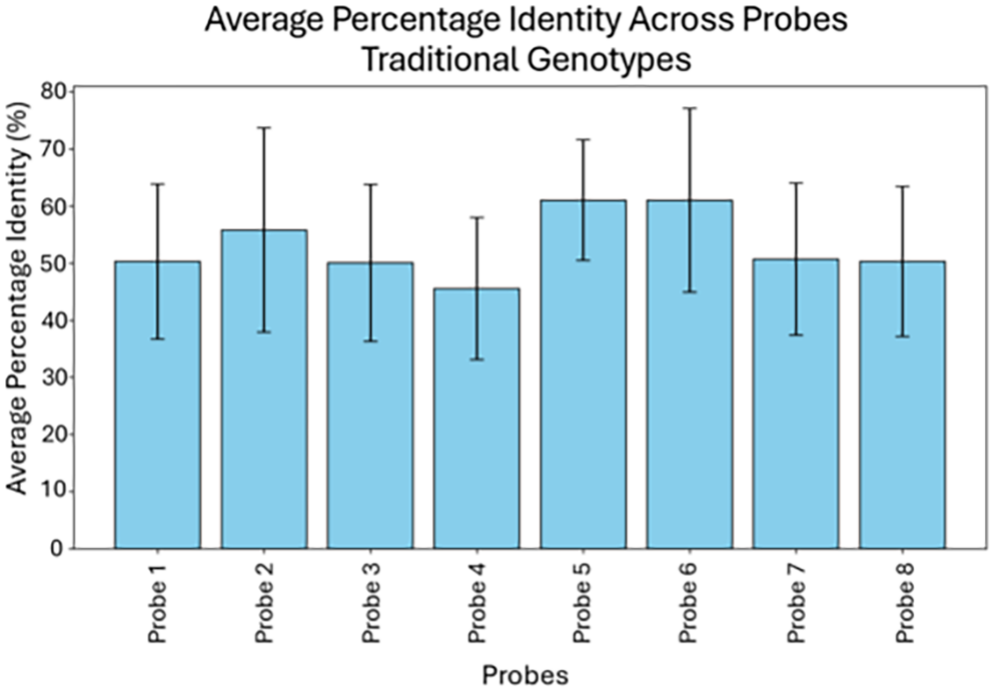
Comparative analysis of probe performance across ASFV p72 genotypes. Bar graphs represent the hybridization efficiency of probes designed for traditional genotypes.

Based on these findings, Table 1 summarizes the results of Clustal Omega’s alignment for each probe, categorizing their binding profiles according to specific genotypes. To standardize binding classifications, percentage identity values were grouped using defined thresholds: strong binding (≥85%), partial binding (60–84%), and weak binding (<60%), consistent with conventional hybridization criteria (35). These classifications were then applied to the mean values to assign strong, partial, or weak binding categories per probe–genotype pair.

**Table 1:** Genotypic Coverage^a^

probe number	base pair length	traditional ASFV genotypes heatmap analysis
1	28	partial binding to genotypes II and XV
2	40	partial binding to genotypes IX and XV
3	50	partial binding to genotypes I, II, and XV
4	60	all genotypes have weak binding
5	60	partial binding to genotypes II, IX, and XV
6	70	partial binding to genotypes II, IX and XV
7	80	all genotypes have weak binding
8	80	all genotypes have weak binding

a Probes were evaluated for genotypic coverage based on heatmap analysis. Binding categories were determined using sequence identity thresholds derived from Clustal Omega alignments. Strong binding: ≥85% sequence identity; Partial binding: 60–84%; Weak binding: <60%.

Probes 1, 2, 3, 5, and 6 exhibited partial binding to at least one genotype, most frequently Genotypes II, IX, and XV. While Probes 4, 7, and 8 showed weak binding across all tested genotypes, indicating reduced hybridization potential and deprioritizing them for downstream validation. Alignment analysis using Clustal Omega demonstrated that probe sequence complementarity occurred exclusively within genotypes I, II, IX, XV, and XXIII. The corresponding heat maps are provided in Appendix B.

Together, the sequence identity matrix, Figure 2, and Table 1 show that Probes 2, 5, and 6 are the most promising candidates. The weak computational performance of Probe 1 aligns with its higher experimental detection limit, highlighting the utility of this screening step.

### Generalized Absorbance Profile of Probe Performance

Representative absorbance spectra illustrate the GNP biosensor function. Figure 3A shows spectral shift from 520 nm with decreasing concentrations of ASFV DNA (4400 to 550 copies), reflecting nanoparticle aggregation. Figure 3B demonstrates specificity with ASFV DNA generating a distinct spectral profile with a peak at 520 nm compared to nontarget bacterial DNA shifting from 520 nm (*E. coli O157*, *S.* Enteritidis, and *S. aureus*). The overlapping curves for nontargets suggest minimal cross-reactivity and strong target discrimination. These data confirm the colorimetric principle of the biosensor, where probe–target hybridization prevents GNP aggregation and maintains the 520 nm peak.

**Figure 3:**
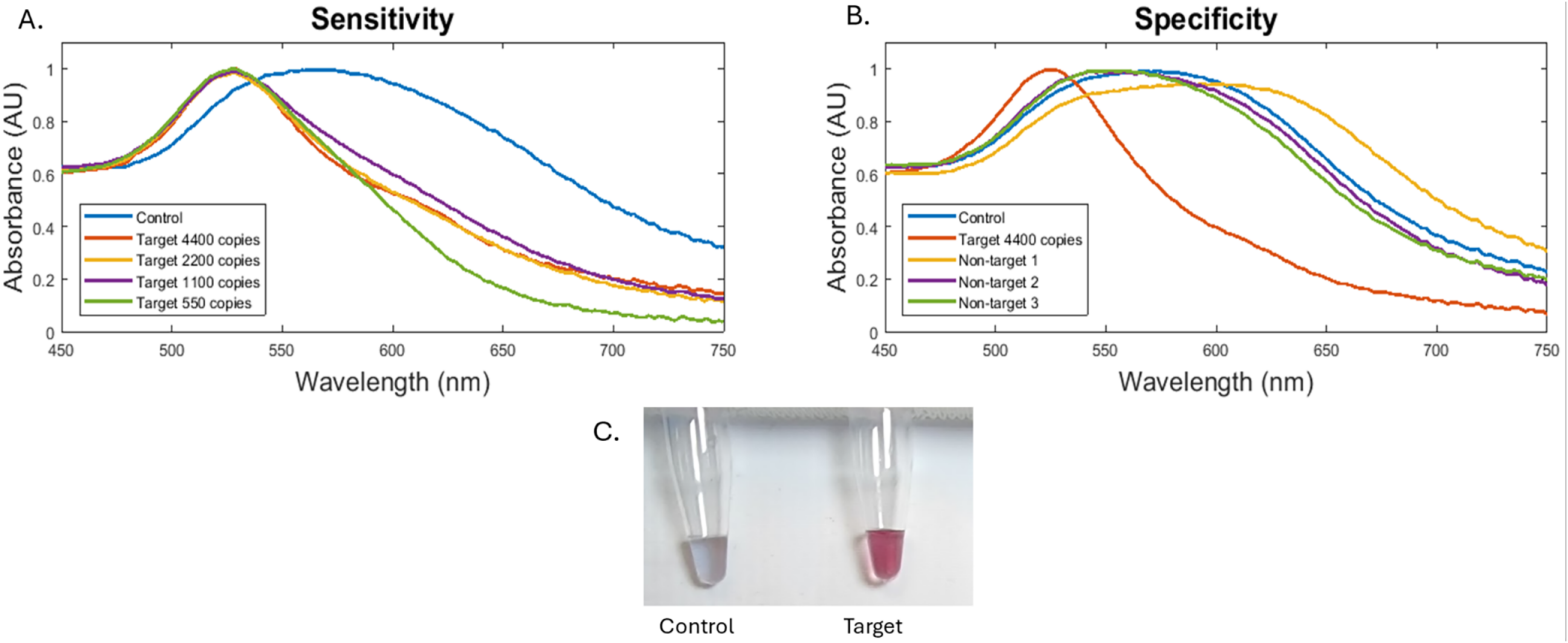
Absorbance spectra illustrating GNP biosensor performance. (A) Sensitivity profile showing spectral changes at decreasing ASFV p72 DNA concentrations (4400 to 550 copies). (B) Specificity profile comparing ASFV target DNA with nontarget bacterial DNA. Spectral shifts confirm discrimination between the target and nontargets through GNP aggregation behavior. (C) Photograph of assay tubes after HCl addition: the control (left) exhibits a gray-blue color due to GNP aggregation in the absence of target DNA, whereas the sample containing ASFV DNA (right) remains red, illustrating the clear, instrument-free visual readout suitable for field deployment.

Figure 3 demonstrates the principle of colorimetric detection measured in the NanoDrop One-C using GNPs, where probe-target hybridization prevents aggregation, resulting in a red color and a peak near 520 nm. In contrast, nonbinding interactions result in aggregation and a shift in absorbance.

### Probe Sensitivity and Specificity Analysis

Following computational probe screening, experimental evaluation was conducted to identify probes with both high specificity and low detection limits for ASFV p72 DNA. Spectrophotometric analysis using the NanoDrop One-C was used to assess probe performance, with statistical comparisons made using two-way ANOVA and Tukey’s multiple comparisons test. The experimental evaluation focused first on specificity to exclude cross-reactive probes, followed by sensitivity to determine the lowest detectable DNA concentration for each probe.

Figure 4 illustrates the specificity analysis, showing the biosensor’s ability to distinguish ASFV p72 DNA from three nontarget bacterial species: *E. coli* O157, *S.* Enteritidis, and *S. aureus*. Each bar represents the difference in mean peak shifts from 520 nm between the control and nontargets for a given probe. Larger differences reflect stronger discrimination and higher specificity. Probes 3, 4, and 6 failed to show significant differentiation (*p* > 0.05) from at least one nontarget organism and were therefore considered nonspecific and eliminated from further analysis. In contrast, Probes 1, 2, 5, 7, and 8 demonstrated clear and statistically significant specificity, validating their selectivity for ASFV DNA.

**Figure 4:**
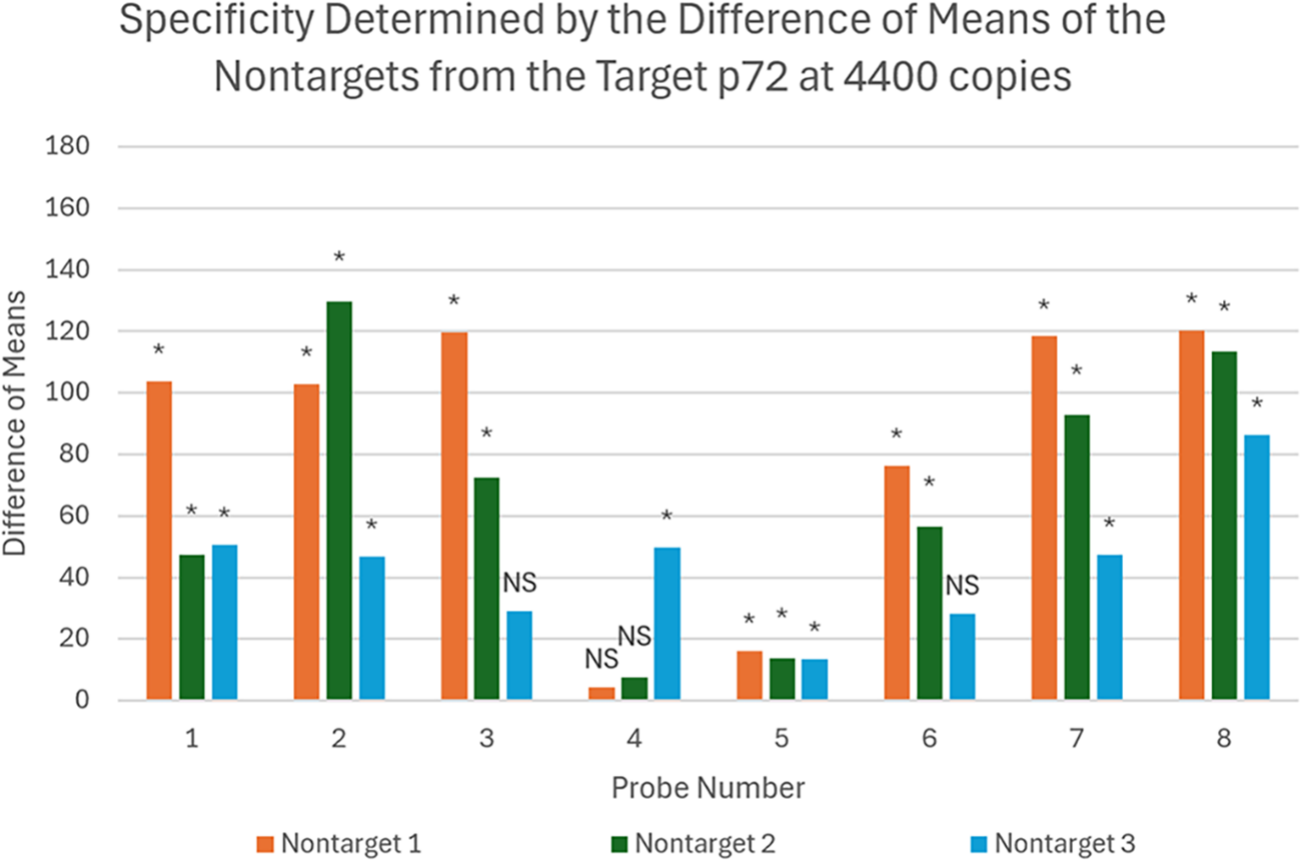
The difference of means of the nontargets from the control determines if the different probes are specific. The difference of means for the eight probes is shown for the nontargets 1–3. The difference of means is the average of the peak shift from 520 nm of each nontarget subtracted from the peak shift average of the control for each probe. The larger the difference in means, the larger the peak shift is, resulting in how gray the color of the biosensor is. It is seen that probes 3, 4, and 6 are not specific (NS). Above the bars are depictions of the significance of the p-values. NS *p* > 0.05; * *p* ≤ 0.05. The experiments for each probe are *n* = 3. The nontarget 1 is *E. coli* O157, nontarget 2 is *S.* Enteritidis, and nontarget 3 is *S. aureus* all at 20 ng/μL.

Figure 5 represents the sensitivity results, which measured the lowest detectable concentration of ASFV p72 DNA for each probe. The biosensor’s colorimetric response was quantified by peak shifts, with larger shifts indicating stronger hybridization. Sensitivity was determined by identifying the lowest DNA concentration at which the biosensor produced a distinct response from the control, with nonoverlapping error bars serving as the threshold for detection. Probes 2 and 5 achieved the highest sensitivity, detecting ASFV DNA at concentrations as low as 550 copies, while Probes 1, 7, and 8 required significantly higher concentrations (≥2200 copies) and were excluded based on insufficient sensitivity for field applications.

**Figure 5:**
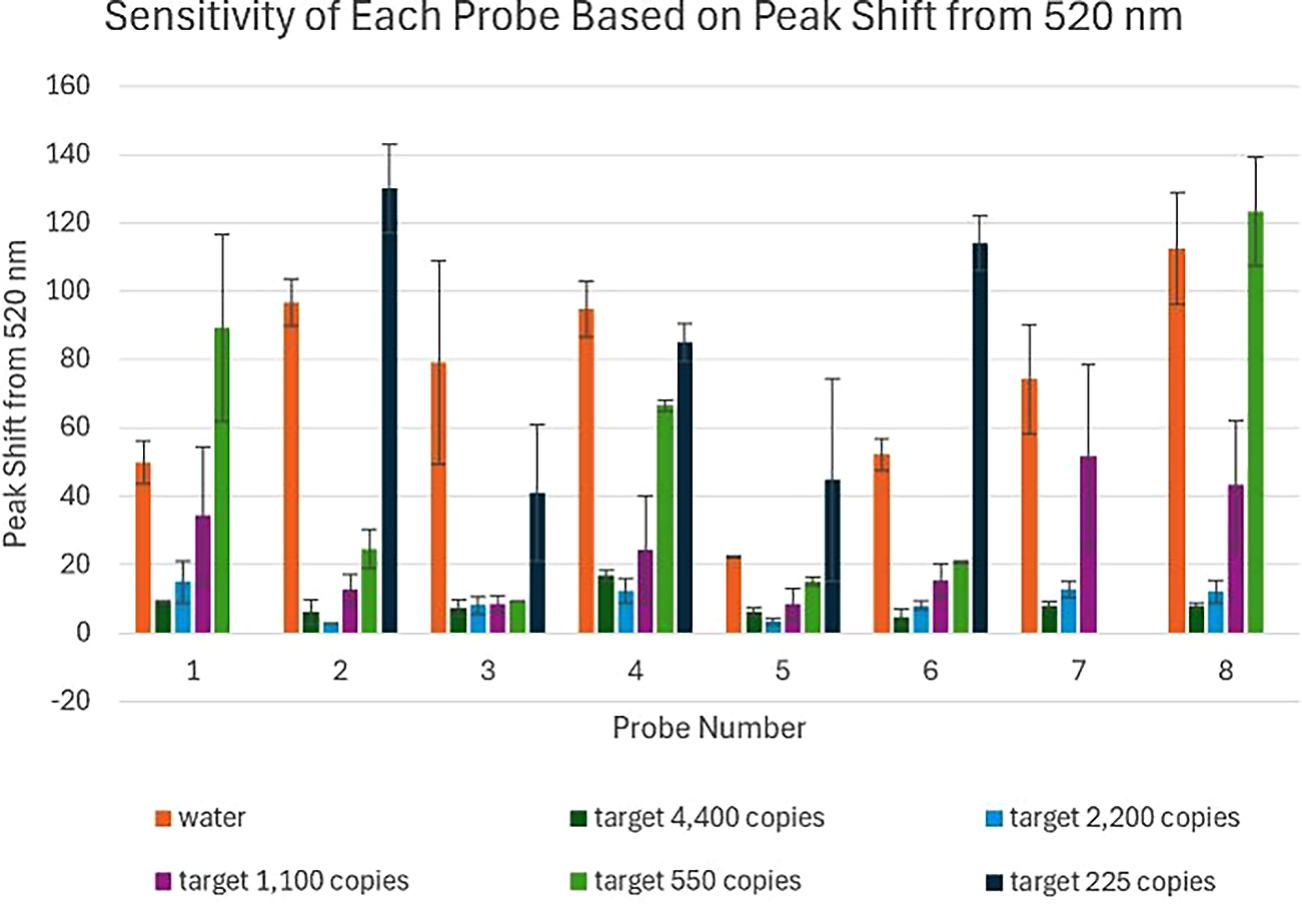
Sensitivity of the probes for the ASFV GNP biosensor. The 8 probes were tested with their probe lengths and the lowest copy number they could detect. The bars are the averages of the *n* = 3 experiments, and the error bars are the standard deviation.

The combined specificity and sensitivity analyses narrowed the optimal probe candidates to Probes 2 and 5. These probes demonstrated both full specificity against nontarget organisms and strong sensitivity at low DNA concentrations. This dual performance makes them suitable for diagnostic use in rapid field-deployable biosensors for ASFV detection. The elimination of Probes 3, 4, and 6 based on cross-reactivity, and Probes 1, 7, and 8 due to high detection thresholds, underscores the necessity of sequential evaluation to identify robust probe candidates.

These findings are summarized in Table 2, emphasizing the importance of optimizing both specificity and sensitivity in biosensor development. A probe must not only reliably detect low concentrations of target DNA but also avoid false positives from unrelated bacterial DNA. Probes 2 and 5 fulfill these criteria and thus offer the most promising profiles for deployment in ASFV surveillance and outbreak response.

**Table 2:** Sensitivity and Specificity of Probes at Five Minutes

probe number	base pair length	specificity	sensitivity (copies)	GC content (%)	melting temperature (°C)
1	28	fully specific	2200	32.5	62.2
2	40	fully specific	550	50.0	67.2
3	50	not specific to nontarget 3	550	50.0	69.5
4	60	not specific to nontargets 1 and 2	550	43.1	68.1
5	60	fully specific	550	54.2	70.7
6	70	not specific to nontarget 3	550	48.1	71.7
7	80	fully specific	2200	41.2	68.6
8	80	fully specific	1100	45.3	71.5

To better understand the superior performance of Probes 2 and 5, their molecular features, including GC content and melting temperature (*T*_m_), were analyzed and results are summarized in Table 2. Probe 2 exhibited a GC content of 50.0% and a *T*_m_ of 67.2 °C, while Probe 5 showed a higher GC content of 54.2% and a *T*_m_ of 70.7 °C. These values suggest that both probes maintain stable duplex formation with target DNA, supporting strong hybridization. In contrast, Probe 1, which showed the weakest sensitivity, had a low GC content of only 32.5%, potentially contributing to its reduced binding strength and higher detection limit. These results align with prior findings that balanced GC content and moderate *T*_m_ are critical for probe efficiency, especially in colorimetric biosensors where rapid hybridization is essential. No significant secondary structures were predicted for Probes 2 and 5, minimizing self-complementarity and steric hindrance. Together, these molecular characteristics explain their high sensitivity and specificity, reinforcing their suitability for ASFV biosensing applications.

### Probe Features and Correlation Analysis

Spearman’s rank correlation was used to evaluate associations between probe features and detection sensitivity (LOD, copies/μL). Among the variables tested, only GC content showed a statistically significant correlation with sensitivity (ρ = −0.80, *p* = 0.016). This negative relationship indicates that probes with higher GC content achieved lower detection thresholds and therefore greater sensitivity (Figure 6). In contrast, probe length (ρ = 0.19, *p* = 0.656), hairpin stability (ρ = −0.35, *p* = 0.388), self-dimer stability (ρ = −0.06, *p* = 0.884), and heterodimer stability (ρ = −0.35, *p* = 0.392) did not show significant associations. These findings suggest that GC content is a stronger determinant of probe performance than length or predicted secondary structures. Full Spearman correlation results, including Δ*G*-based parameters, are provided in Table S2.

**Figure 6:**
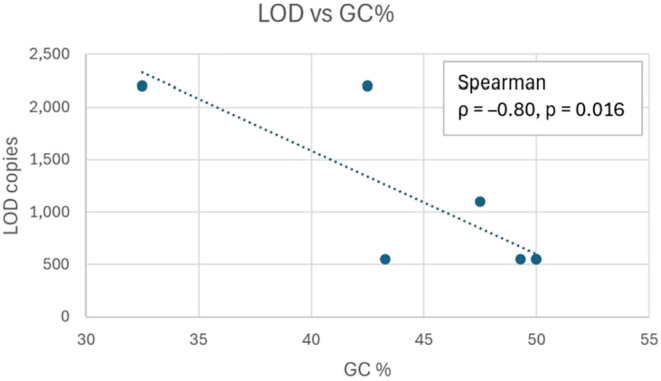
Relationship between GC content and analytical sensitivity of the ASFV biosensor. Scatterplot of probe GC content (%) versus detection limit (LOD, copies/μL). A significant negative correlation was observed (Spearman ρ = −0.80, *p* = 0.016), showing that probes with higher GC content achieved lower detection thresholds and greater sensitivity.

Figure 6 illustrates the negative correlation between GC content and LOD. Although Δ*G*-based stability parameters were examined, they did not predict sensitivity, suggesting sequence composition is a more influential determinant than probe length or predicted structure. The Spearman correlation coefficient (ρ = −0.80, *p* = 0.016) supports this observation, confirming that sequence composition plays a more influential role than probe length or secondary structure predictions. This nonparametric test, calculated using eq 1, does not assume linearity

1 $$\rho =1-\frac{6\sum d_{i}^{2}}{n\left(n^{2}-1\right)}$$

where ρ is the Spearman coefficient, *d_i_* is the difference in ranks for each data pair, and *n* is the total number of observations.

### Extended Sensitivity Assessment at 10 min

To further validate the robustness of the biosensor and assess whether probe performance remained effective beyond the initial 5 min detection window, additional sensitivity testing was conducted at a 10 min incubation period. This analysis focused on Probes 2 and 5, which previously demonstrated the best balance of sensitivity and specificity in both Clustal Omega binding and experimental assays.

Figure 7 illustrates the peak shift from 520 nm for serial dilutions of synthetic ASFV p72 DNA tested with Probes 2 and 5 at the 10 min time point. Probes 2 and 5 also maintained their specificity to ASFV. Shown in Figure 7, both probes retained their sensitivity, exhibiting clear differentiation between water controls and ASFV DNA at multiple concentration levels. Both Probe 2 and 5 maintained detection capabilities down to 550 copies, which remained statistically distinguishable from the control (water).

**Figure 7:**
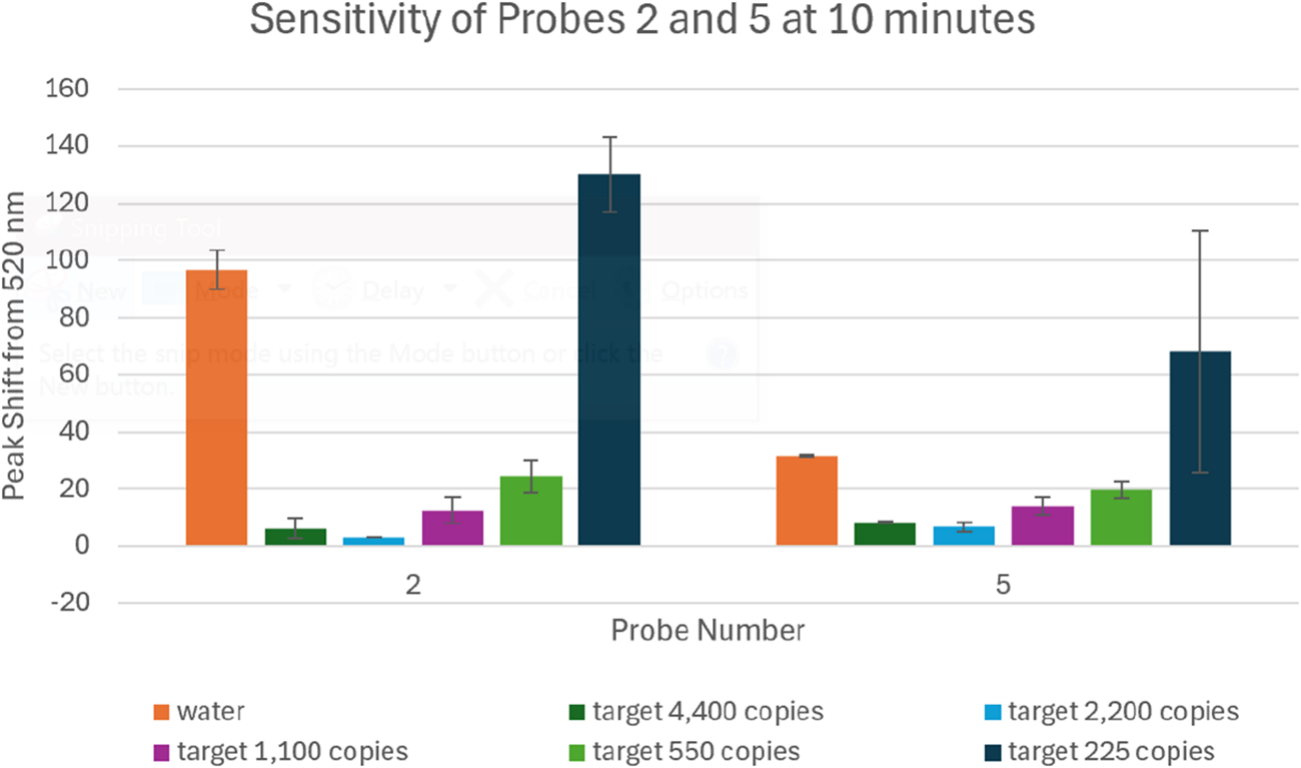
Peak shift from 520 nm is shown for varying concentrations of ASFV p72 DNA (4400 to 225 copies) using Probes 2 and 5 after a 10 min incubation. Water served as a negative control. Error bars represent standard deviations across *n* = 3 replicates.

This extended analysis reinforces the temporal stability and sustained hybridization of these two probes, confirming that their diagnostic performance is not limited to the short 5 min reaction time. These results support the feasibility of using the biosensor in field conditions where slight deviations from the optimal 5 min window may occur, ensuring consistent ASFV detection in real-world scenarios. Together with previous findings, this analysis confirms that Probes 2 and 5 remain the most viable candidates for ASFV detection, offering rapid, sensitive, and reliable detection even after extended incubation.

## Discussion

The development of a GNP-based biosensor for the detection of ASFV presents a promising avenue for rapid, field-deployable diagnostics. This study evaluated the performance of eight oligonucleotide probes targeting the ASFV p72 gene, integrating computational and experimental analyses to optimize probe selection based on sensitivity, specificity, and genotypic coverage.

Initial in silico validation using Clustal Omega confirmed that all eight probes displayed at least partial complementarity to one or more ASFV genotypes, supporting their use in downstream testing. Heatmap and bar plot analyses of sequence identity values revealed that Probes 2, 5, and 6 exhibited higher average percentage identity values, particularly across Genotypes II, IX, and XV. In contrast, Probes 4, 7, and 8 displayed weak binding across all genotypes, highlighting their limited hybridization potential.

The observed alignment of probes to ASFV genotypes I, II, IX, XV, and XXIII is notable, since these genotypes include both globally and regionally important strains. Genotype II, which emerged in Georgia in 2007, has since become the dominant lineage across Europe and Asia and was detected in the Caribbean (Dominican Republic and Haiti) in 2021.^30,43^ Meanwhile, genotypes II, IX, X, XV, and XVI remain prevalent in sub-Saharan Africa, particularly in Tanzania.^44,45^ This suggests that the biosensor in this study effectively covers strains most relevant to current outbreaks, though additional probe designs may be required for full coverage across all 24 p72 genotypes.

Experimental specificity testing using nontarget bacterial DNA revealed that Probes 3, 4, and 6 cross-reacted, disqualifying them from further consideration. Meanwhile, Probes 1, 2, 5, 7, and 8 maintained full specificity. Sensitivity assessments confirmed that Probes 2, 3, 4, 5, and 6 could detect ASFV DNA at a threshold of 550 copies. However, Probes 1, 7, and 8 required significantly higher concentrations (1100–2200 copies) for detectable signal generation, suggesting weaker hybridization efficiency or slower kinetic response. Among the high-performing candidates, Probes 2 and 5 consistently combined strong sensitivity with robust specificity, making them optimal choices for further validation. Additional experimental validation showed that Probes 2 and 5 were effective at 10 min reaction times, further enhancing their field applicability. These probes demonstrated clear peak shifts at low DNA concentrations (down to 550 copies), suggesting potential for early detection in real-world surveillance scenarios.

Statistical analysis provided additional insight into probe performance. Probe length, hairpin stability, and dimerization energies showed no significant relationship with sensitivity, while Spearman’s correlation (Table S2) identified GC content as the only feature significantly associated with performance (ρ = −0.80, *p* = 0.016). Probes with higher GC content achieved lower detection thresholds, aligning with established principles that balanced GC content stabilizes probe–target duplexes without excessive secondary structure. These results indicate that sequence composition is a more reliable predictor of sensitivity than either probe length or structural predictions.

While midlength probes performed well experimentally, their effectiveness appears to stem from favorable GC content rather than size. This aligns with established principles of nucleic acid hybridization, where optimal GC content stabilizes probe–target duplexes without introducing excessive secondary structure. For a rapid, colorimetric biosensor, maintaining this balance is critical to achieving both sensitivity and speed. Together, the combination of in silico alignments, experimental assays, and correlation analysis supports the conclusion that GC content is a key driver of probe performance in nanoparticle-based ASFV biosensors. This provides a rational design criterion for selecting and optimizing probes in future diagnostic platforms.

The binding advantage was also considered to assess whether probes preferentially hybridized with the target over self-structures. However, this metric did not align with sensitivity outcomes. For example, Probe 1 exhibited the highest binding advantage yet required 2200 copies for detection, while Probes 2 and 5 had modest or slightly unfavorable binding advantage values but achieved detection at 550 copies. This discrepancy suggests that binding advantage, although conceptually useful, oversimplifies the interplay of hybridization kinetics, steric hindrance, and nanoparticle surface effects. By contrast, GC content consistently correlated with performance, reinforcing its importance as a design criterion for biosensors.

The current biosensor was optimized at pH 8.0 and requires two temperature steps, 95 °C for denaturation and 55 °C for hybridization. While this design limits portability compared with isothermal systems, it eliminates the need for enzyme reagents that add cost and require cold storage. To improve usability in low-resource settings, a hand-held thermocycler or dual water bath system maintained at 95 and 55 °C could replace the lab-based thermocycler, providing precise temperature control without complex instrumentation. Because sample matrices such as feed, feces, and swabs often vary in pH, assay performance can be stabilized through tailored buffer systems. Tris-HCl or phosphate buffers can be used to adjust acidic matrices such as feces or feed extracts toward neutral conditions, while HEPES or MOPS buffers may help stabilize slightly alkaline samples such as oral fluids. These buffering strategies will help preserve nanoparticle stability and hybridization fidelity across diverse sample types without excessive dilution of the target.

In terms of stability, the GNPs remained functional for over a year at 4 °C and tolerated short-term transport at room temperature. Extended studies on shelf life, freeze–thaw tolerance, and performance under diverse environmental conditions remain necessary. GNPs are considered biocompatible at the concentrations used here, but waste management should account for heavy metal disposal.

While the biosensor showed promising in vitro results, we acknowledge that the platform has not yet undergone field validation using clinical ASFV samples. Due to strict biosecurity and import restrictions in the United States, access to authentic ASFV material was not possible; thus this study was limited to synthetic DNA. As ASFV is a foreign animal disease, research involving live or infectious ASFV material must be conducted in high-containment biosafety level-3 laboratories and is regulated under federal oversight. This constraint limits the feasibility of performing field validation domestically. Nonetheless, the diagnostic framework aligns with core principles outlined by the World Organisation for Animal Health (WOAH) for assay development, including analytical sensitivity, specificity, and cross-reactivity testing using surrogate organisms.

Early stage validation of ASFV biosensors typically begins with synthetic targets to benchmark assay chemistry, readout, and workflow before progressing to complex matrices or clinical specimens. For instance, Zhao et al. quantified the performance of a CRISPR/Cas14–G-quadruplex DNAzyme paper test using synthetic ASFV DNA without field testing, illustrating an accepted first step toward translation.^46^ Similarly, a one-pot CRISPR-Cas12a visual workflow has been reported to have analytical sensitivity using laboratory-prepared DNA targets within a single-tube reaction, prior to real-world deployments.^47^

Although this study used synthetic ASFV DNA, this approach reflects common practice in early stage biosensor development. For example, a GNP^48^ and electrochemical DNA-based^49^ biosensors for Zika virus, GNP-based biosensors for dengue virus,^50^ and deoxyribozyme biosensor for Nipah virus^51^ were also initially validated with synthetic nucleic acids before progression to clinical material. Such staged validation is widely accepted for proof-of-concept studies of zoonotic viral biosensors.

In line with this staged pathway, the study establishes the analytical foundation with synthetic ASFV DNA and reserves matrix-specific optimization and clinical validation for subsequent work. Future work will include validation using field specimens such as blood, swabs, or environmental samples through international collaborations in ASFV-endemic regions to assess real-world diagnostic performance.

Comparison with other diagnostic platforms highlights the balance of sensitivity, speed, cost, and portability. Real-time PCR achieves ∼10 copies per reaction but requires 1.5–2 h, expensive equipment, and trained personnel.^52^ LAMP detects ∼6 copies/μL in ∼60 min,^53^ while CRISPR–RPA achieves single-copy sensitivity in 30–45 min but requires specialized enzymes and lateral-flow strips.^54^ Commercial antigen lateral-flow tests deliver results in 15–30 min at low cost but detect only high viral loads with ∼65–68% sensitivity.^55^ By contrast, the GNP‑based biosensor in this paper requires a single 30 min thermocycler incubation, standard extraction (5 USD per sample), and gold nanoparticles (1 USD per assay) to detect approximately 550 copies with a naked-eye colorimetric readout, offering fast, cheaper and more portable performance than the compared assays while remaining simpler and more field-deployable than qPCR.

The GNP biosensor provides a rapid naked-eye red-to-blue readout, subjective color perception can vary among users. In this study, color changes were quantified using NanoDrop spectra by measuring the peak shift from 520 nm. To improve field applicability, smartphone-based imaging and color-analysis tools could be integrated to extract red, green, blue (RGB) or hue-saturation-value (HSV) values. Similar approaches have been shown to standardize visual thresholds, minimize the effect of lighting variability, and provide reliable field-ready quantification.^56–60^

Overall, the integration of sequence alignment, genotypic analysis, experimental testing, and statistical modeling allowed for a robust and multidimensional evaluation of probe performance. This study establishes probes 2 and 5 as strong candidates for ASFV detection and highlights the importance of combining computational prediction with empirical validation in biosensor development. Future efforts should focus on clinical validation, environmental stability testing, and integration into portable diagnostic formats.

## Conclusion

This study demonstrates the feasibility and effectiveness of a GNP-based biosensor for rapid and specific detection of ASFV. Among the eight tested oligonucleotide probes targeting the p72 gene, probes 2 and 5 emerged as the most robust candidates, showing strong genotypic coverage, low detection limits, and high specificity. The integration of sequence alignment, spectrophotometric validation, and statistical modeling enabled a rigorous evaluation framework that can inform future biosensor design.

Statistical analysis revealed that GC content, rather than probe length, predicted secondary structures, or calculated binding advantage, was the strongest determinant of probe performance. Probes with higher GC content achieved lower detection thresholds, underscoring the importance of sequence composition in biosensor design. While binding advantage and Δ*G*-based stability metrics offered valuable theoretical insight, they did not reliably align with sensitivity outcomes in this data set.

Importantly, this platform achieved target DNA detection in as little as 5 min, with performance maintained at 10 min, highlighting its suitability for field deployment. By eliminating nonspecific and low-performing probes early in the design process, this work underscores the importance of combining computational and experimental screening to optimize diagnostic tools. With further validation using clinical samples, this biosensor holds strong potential as a rapid, accessible, and scalable solution for ASFV surveillance, especially in high-risk or resource-limited settings.

Future work should focus on validating the biosensor with clinical ASFV samples to assess its performance in real-world scenarios. Additional research should also explore probe adaptability to emerging ASFV genotypes to ensure sustained diagnostic reliability. The integration of this biosensor into routine surveillance programs could provide early detection capabilities, particularly in regions vulnerable to ASFV introduction. By reducing diagnostic turnaround time and increasing accessibility, this platform has the potential to enhance global ASFV biosecurity and facilitate timely outbreak interventions.

## Supplementary Material



## Notes

Results of two-way ANOVA with Tukey’s posthoc comparisons for each probe; comparisons between controls, serial dilutions, nontarget controls; significant differences (*p* < 0.05) (Table S1); Spearman’s rank correlations for probe features and analytical sensitivity; statistical summary for GC content and other probe parameters (Table S2); Python code for statistical analysis and heatmap generation (Appendix A); heatmaps for traditional genotypes (Appendix B) (PDF)
