# Supplementary material for: Proof-of-Concept Nanoparticle-Based Biosensor for Detecting the African Swine Fever Virus Across Multiple Genotypes Using In Silico and In Vitro Approaches
Source: ACS Omega. 2025 Oct 22;10(43):51028–38. doi: 10.1021/acsomega.5c05375 (PMC12593959; doi:10.1021/acsomega.5c05375)
Supplement: Supplementary file 1 [file ao5c05375_si_001.pdf]

Supporting Information:

Proof-of-Concept Nanoparticle-Based Biosensor for Detecting the African Swine Fever Virus Across Multiple Genotypes Using In Silico and In Vitro Approaches

Chelsie Boodoo[1], and Evangelyn C. Alocilja[1]*

1 Department of Biosystems and Agricultural Engineering, Michigan State University, East Lansing, Michigan, United States

* Correspondence: alocilja@msu.edu (EC.); Tel.: +1-517-432-8672

Table S1

Probe #	Comparison	Difference of Means	Degrees of Freedom (df)	p-value
1	Target 4400 copies x NT1	103.83	(7,16)	1.04E-06
1	Target 4400 copies x NT2	47.50	(7,16)	1.02E-03
1	Target 4400 copies x NT3	50.67	(7,16)	6.10E-04
1	Control x Target 4400 copies	40.6667	(7,16)	3.27E-03
1	Control x Target 2200 copies	34.8333	(7,16)	1.20E-01
1	Control x Target 1100 copies	15.5	(7,16)	7.46E-01
1	Control x Target 550 copies	11.4	(7,16)	8.12E-01
2	Target 4400 copies x NT1	102.83	(8, 18)	3.66E-08
2	Target 4400 copies x NT2	129.83	(8, 18)	4.15E-08
2	Target 4400 copies x NT3	46.67	(8, 18)	4.46E-04
2	Control x Target 4400 copies	90.5	(8, 18)	1.20E-06
2	Control x Target 2200 copies	93.6667	(8, 18)	6.67E-07
2	Control x Target 1100 copies	84	(8, 18)	6.50E-08
2	Control x Target 550 copies	72.1667	(8, 18)	7.74E-06

2	Control x Target 225 copies	33.3333	(8, 18)	7.54E-01
3	Target 4400 copies x NT1	119.70	(8, 18)	1.68E-11
3	Target 4400 copies x NT2	72.55	(8, 18)	1.57E-06
3	Target 4400 copies x NT3	29.15	(8, 18)	1.11E-01
3	Control x Target 4400 copies	71.7	(8, 18)	1.54E-03
3	Control x Target 2200 copies	70.6125	(8, 18)	7.13E-09
3	Control x Target 1100 copies	69.55	(8, 18)	1.13E-10
3	Control x Target 550 copies	13.42	(8, 18)	6.82E-01
3	Control x Target 225 copies	7.36	(8, 18)	8.54E-01
4	Target 4400 copies x NT1	4.33	(8, 18)	9.68E-01
4	Target 4400 copies x NT2	7.50	(8, 18)	8.17E-01
4	Target 4400 copies x NT3	49.67	(8, 18)	9.50E-04
4	Control x Target 4400 copies	78	(8, 18)	1.08E-05
4	Control x Target 2200 copies	25.3333	(8, 18)	2.60E-04
4	Control x Target 1100 copies	22.5	(8, 18)	6.85E-04

4	Control x Target 550 copies	38.6667	(8, 18)	6.20E-06
4	Control x Target 225 copies	8.2	(8, 18)	7.64E-01
5	Target 4400 copies x NT1	16.17	(8, 18)	1.51E-05
5	Target 4400 copies x NT2	13.83	(8, 18)	6.05E-05
5	Target 4400 copies x NT3	13.33	(8, 18)	8.34E-05
5	Control x Target 4400 copies	0.1667	(8, 18)	1.00E+00
5	Control x Target 2200 copies	13.6667	(8, 18)	1.54E-04
5	Control x Target 1100 copies	7	(8, 18)	2.11E-02
5	Control x Target 550 copies	20.90	(8, 18)	3.97E-01
5	Control x Target 225 copies	9.40	(8, 18)	7.35E-01
6	Target 4400 copies x NT1	76.33	(8, 18)	1.11E-04
6	Target 4400 copies x NT2	56.50	(8, 18)	1.30E-03
6	Target 4400 copies x NT3	28.17	(8, 18)	9.45E-02
6	Control x Target 4400 copies	48	(8, 18)	4.35E-03
6	Control x Target 2200 copies	29.1667	(8, 18)	1.01E-01

6	Control x Target 1100 copies	21.8333	(8, 18)	3.07E-01
6	Control x Target 550 copies	16.3333	(8, 18)	5.90E-01
6	Control x Target 225 copies	76.8333	(8, 18)	7.82E-01
7	Target 4400 copies x NT1	118.3333	(6,14)	2.00E-06
7	Target 4400 copies x NT2	92.8333	(6,14)	1.88E-05
7	Target 4400 copies x NT3	47.5	(6,14)	4.50E-03
7	Control x Target 4400 copies	66.3333	(6,14)	3.47E-04
7	Control x Target 2200 copies	61.6667	(6,14)	5.93E-03
7	Control x Target 1100 copies	22.6667	(6,14)	3.55E-01
8	Target 4400 copies x NT1	120.17	(7,16)	1.46E-07
8	Target 4400 copies x NT2	113.50	(7,16)	2.46E-07
8	Target 4400 copies x NT3	86.33	(7,16)	3.21E-06
8	Control x Target 4400 copies	104.5	(7,16)	5.30E-07
8	Control x Target 2200 copies	100.3333	(7,16)	4.58E-05
8	Control x Target 1100 copies	69	(7,16)	6.65E-04

8	Control x Target 550 copies	8.9	(7,16)	7.49E-01

Table S1: Results of two-way ANOVA with Tukey's post-hoc multiple comparisons for each probe. Comparisons were made between control reactions, target DNA at serial dilutions (4400 to 225 copies), and three non-target controls (NT1 *E. coli,* NT2 *S.* enteritdis, NT3 *S. aureus*). Values are reported as difference of means with associated degrees of freedom (df) and p-values. Significant differences ($p < 0.05$).

Table S2

Probe	Length	GC %	Hairpin ΔG min (kcal/mol)	Self-dimer ΔG min (kcal/mol)	Hetero-dimer ΔG min (kcal/mol)	Hairpin Tm Max (ºC)	Duplex Tm (ºC)	Binding advantage (ΔΔG adv) = |ΔG hetero dimer| - |ΔG self dimer|	LOD copies
1	28	32.5	-0.63	-3.9	-19.44	32.2	71.3	15.54	2,200
2	40	50	-0.2	-6.37	-5.02	59.4	74.7	-1.35	550
3	50	50	0.67	-8.26	-6.21	47.9	77.1	-2.05	550
4	60	43.3	-1.54	-13.74	-6.68	63.9	76.3	-7.06	550
5	60	50	-0.46	-6.91	-9.82	58.9	78.3	2.91	550
6	70	49.3	-0.2	-9.75	-9.75	59.4	79.6	0	550
7	80	42.5	-1.54	-13.74	-6.68	63.9	76.9	-7.06	2,200
8	80	47.5	0.29	-10.23	-8.09	53.6	79.4	-2.14	1,100

Table S2: Spearman's rank correlation was calculated using probe features and analytical sensitivity (LOD, copies/µL). Independent variables included probe length, GC content, hairpin stability (ΔG), self-dimer stability (ΔG), and hetero-dimer stability (ΔG). Only GC content demonstrated a statistically significant association with sensitivity ($\rho = -0.80$, $p = 0.016$), while all other features showed no significant correlations.

Appendix A: Python Code for Statistical and Visualization Analysis

Heatmap Generation for Genotypic Hybridization Efficiency

```
import pandas as pd

import seaborn as sns

import matplotlib.pyplot as plt

import os

file_name = "31Genotypes2.xlsx"

output_dir = "heatmaps_combined"

os.makedirs(output_dir, exist_ok=True)

probe_columns = ["Probe 1", "Probe 2", "Probe 3", "Probe 4",
```

```
        "Probe 5", "Probe 6", "Probe 7", "Probe 8"]
df = pd.read_excel(file_name)
df[probe_columns] = df[probe_columns].apply(pd.to_numeric, errors="coerce")
df = df.dropna(subset=probe_columns, how='all')
heatmap_data = df[["Genome Accession Number"] +
probe_columns].drop_duplicates().set_index("Genome Accession Number")
plt.figure(figsize=(24, 18))
heatmap = sns.heatmap(
  heatmap_data,
  annot=True,
  fmt=".1f",
  cmap="coolwarm",
  cbar_kws={"label": "Percentage Identity (%)"},
  xticklabels=probe_columns,
  yticklabels=heatmap_data.index
)

plt.title("Heatmap for ASFV Genotypic Hybridization", fontsize=30)
plt.xlabel("Probes", fontsize=24)
plt.ylabel("Genome Accession Numbers", fontsize=24)
output_file = os.path.join(output_dir, f"{file_name.replace('.xlsx', '')}_heatmap.png")
plt.savefig(output_file, bbox_inches="tight")
plt.close()
print(f"Heatmap saved as '{output_file}'!")
```

Bar Graphs

```
import pandas as pd
import seaborn as sns
import matplotlib.pyplot as plt
import os
```

```
file_name = "31genomes.xlsx"
output_dir = "plots_new_genotypes"
os.makedirs(output_dir, exist_ok=True)
probe_columns = ["Probe 1", "Probe 2", "Probe 3", "Probe 4",
                 "Probe 5", "Probe 6", "Probe 7", "Probe 8"]

df = pd.read_excel(file_name)
df[probe_columns] = df[probe_columns].apply(pd.to_numeric, errors="coerce")
probe_means = df[probe_columns].mean()
probe_stds = df[probe_columns].std()
plt.figure(figsize=(10, 6))
plt.bar(probe_means.index, probe_means.values,
        yerr=probe_stds.values, capsize=5,
        color="skyblue", edgecolor="black")
plt.title("Average Percentage Identity Across Probes", fontsize=14)
plt.ylabel("Average % Identity", fontsize=12)
plt.xlabel("Probes", fontsize=12)
plt.xticks(rotation=45)
plt.tight_layout()
plt.savefig(os.path.join(output_dir, "bar_plot_probes.png"), dpi=300)
plt.show()
```

Appendix B: Heatmaps for Traditional Genotypes

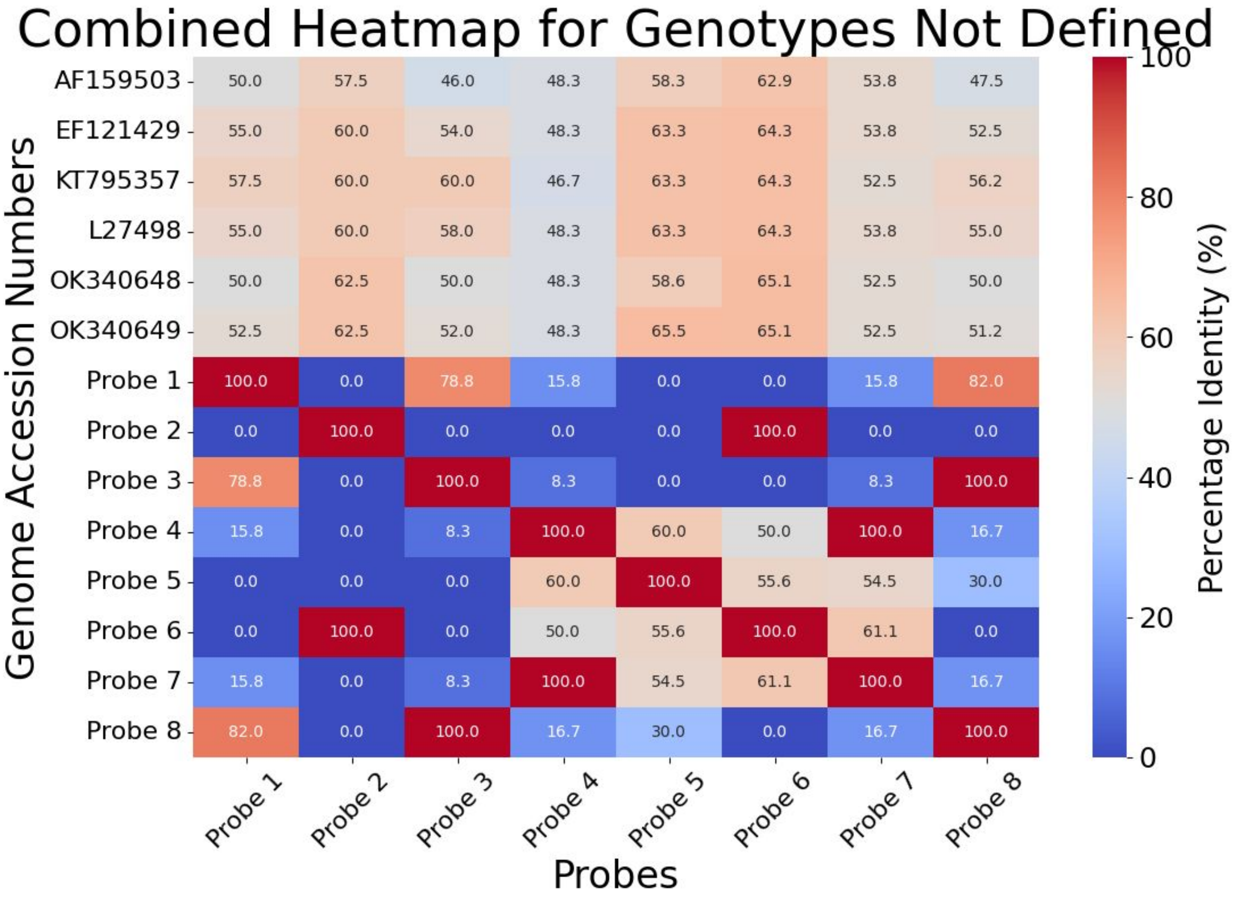

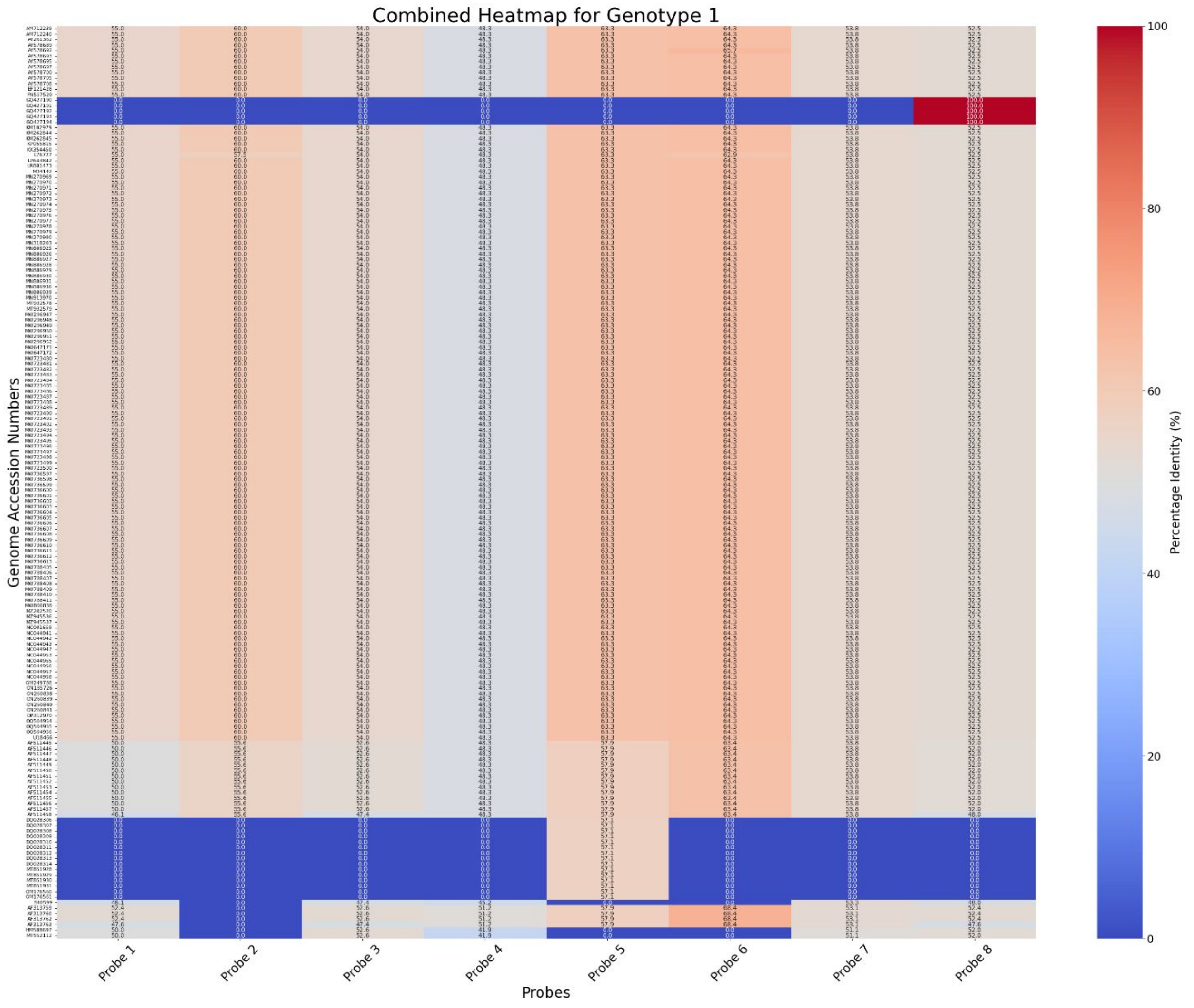

Combined Heatmap for Genotype 1
Genome Accession Numbers
Probe 1
Probe 2
Probe 3
Probe 4
Probe 5
Probe 6
Probe 7
Probe 8
Probes
Percentage Identity (%)
100
80
60
40
20
0

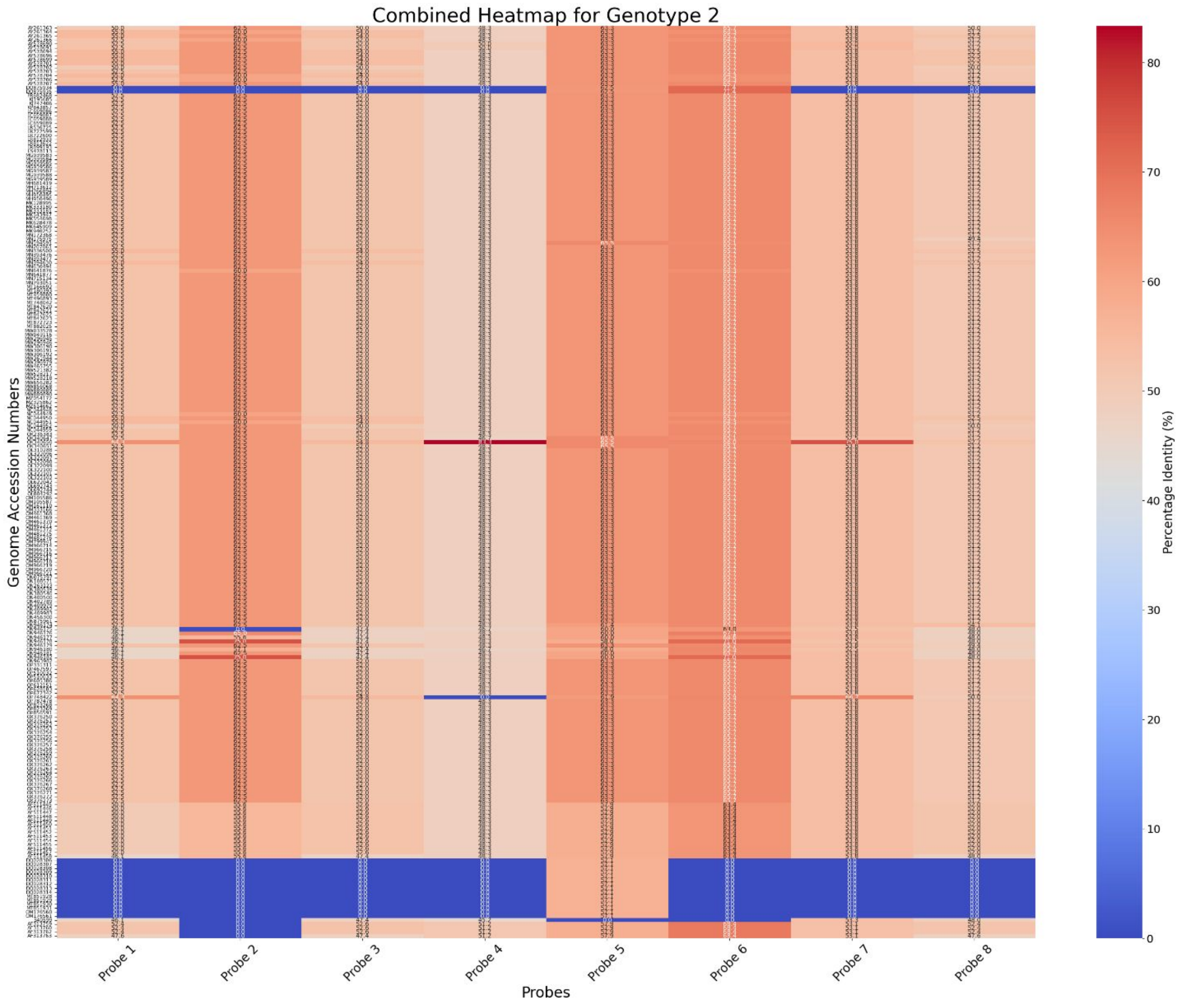

Combined Heatmap for Genotype 2
Genome Accession Numbers
Probe 1
Probe 2
Probe 3
Probe 4
Probe 5
Probe 6
Probe 7
Probe 8
Probes
Percentage Identity (%)
80
70
60
50
40
30
20
10
0

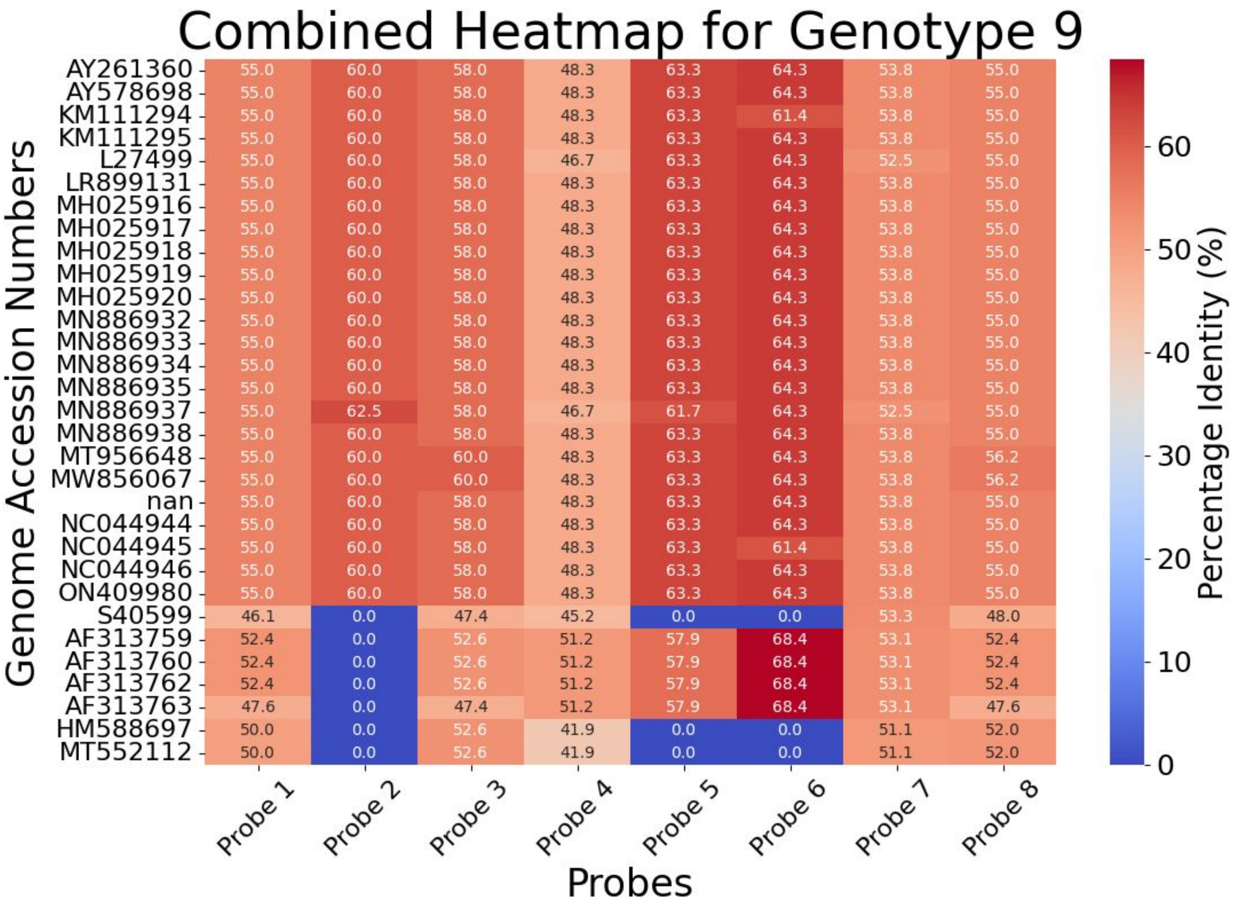

Combined Heatmap for Genotype 9
Genome Accession Numbers
Probes
Percentage Identity (%)

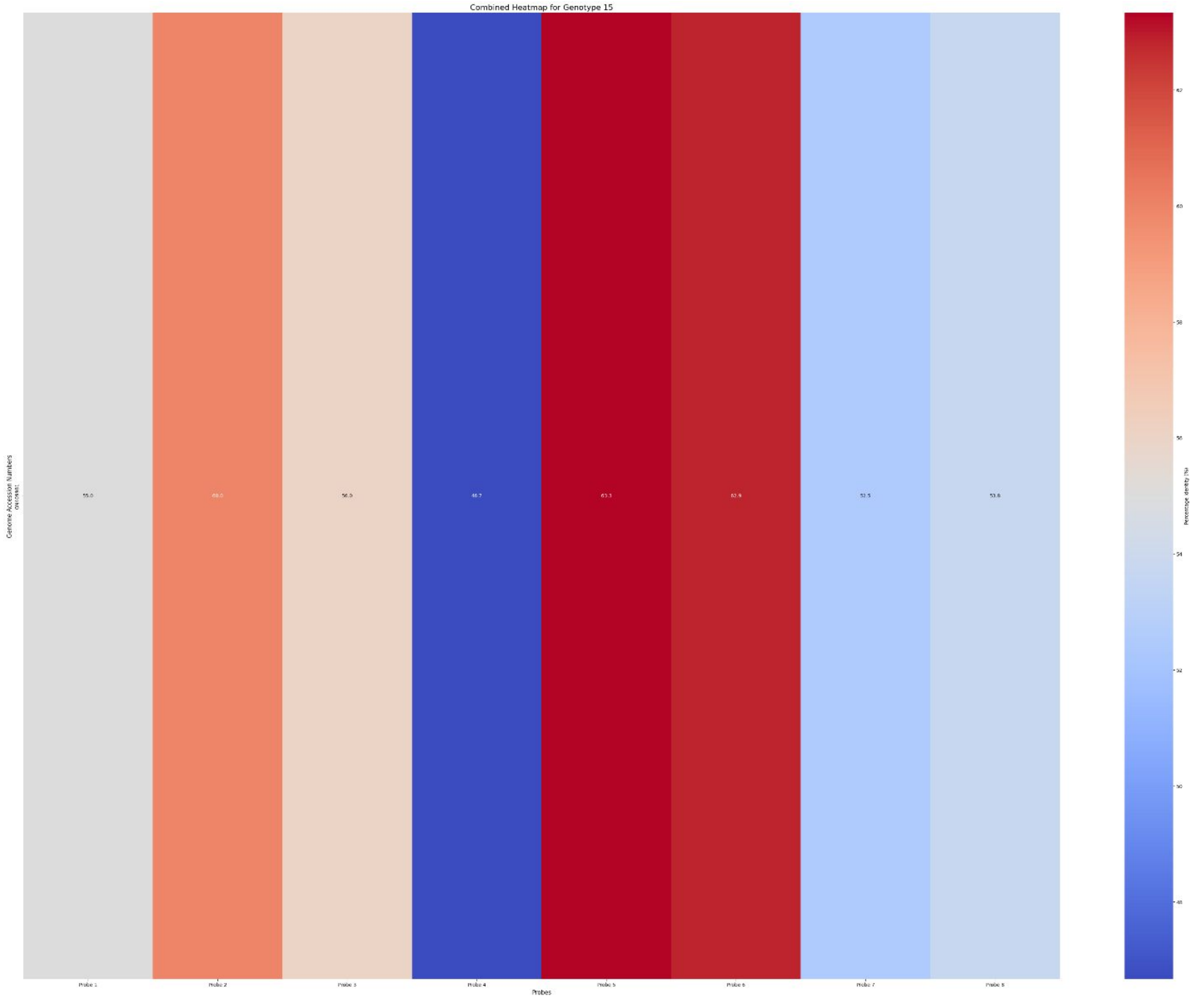
Combined Heatmap for Genotype 15
Genome Accession Numbers
55.0
60.0
56.0
46.7
63.3
62.9
52.5
53.8
Probe 1
Probe 2
Probe 3
Probe 4
Probe 5
Probe 6
Probe 7
Probe 8
Probes
Percentage Identity (%)
62
60
58
56
54
52
50
48

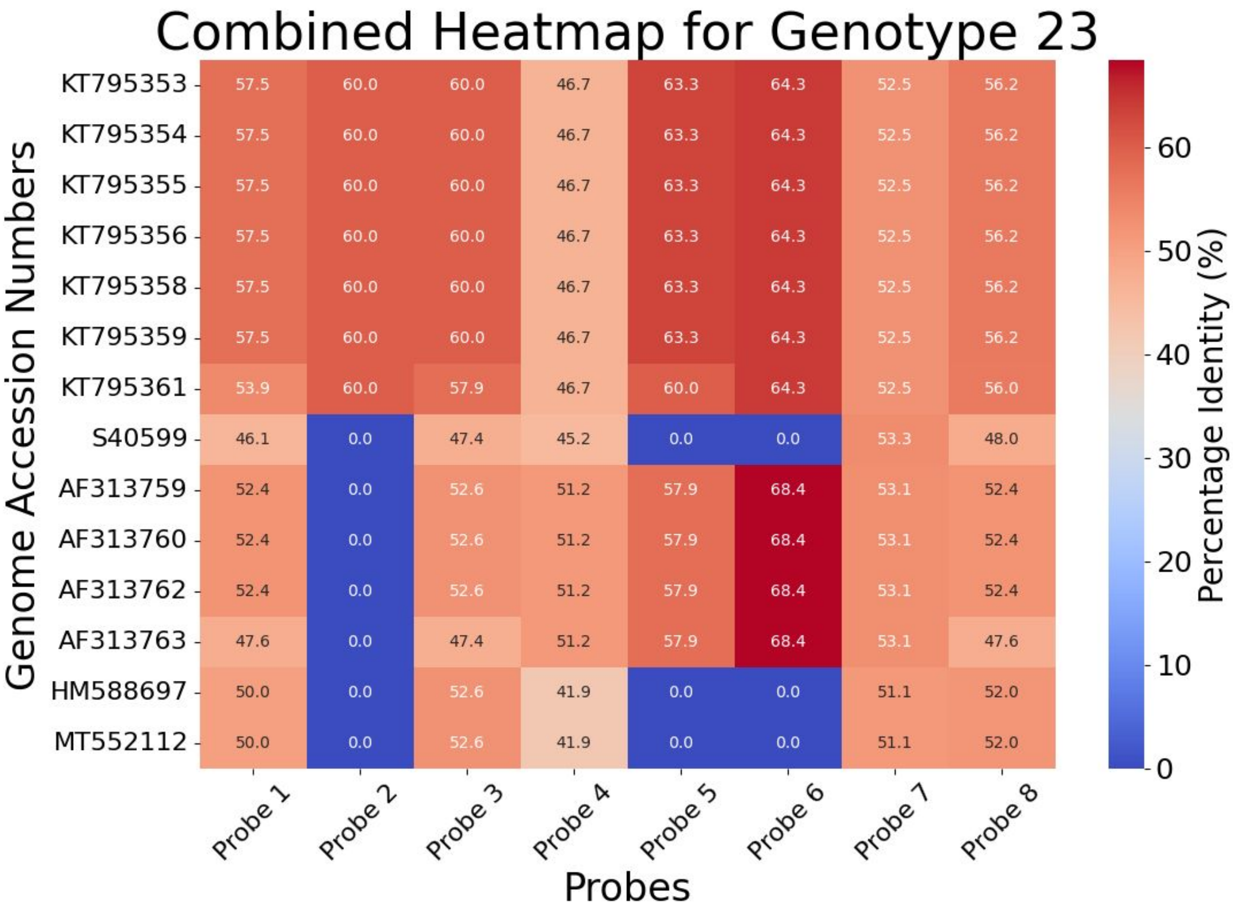
Combined Heatmap for Genotype 23
Genome Accession Numbers
KT795353 57.5 60.0 60.0 46.7 63.3 64.3 52.5 56.2
KT795354 57.5 60.0 60.0 46.7 63.3 64.3 52.5 56.2
KT795355 57.5 60.0 60.0 46.7 63.3 64.3 52.5 56.2
KT795356 57.5 60.0 60.0 46.7 63.3 64.3 52.5 56.2
KT795358 57.5 60.0 60.0 46.7 63.3 64.3 52.5 56.2
KT795359 57.5 60.0 60.0 46.7 63.3 64.3 52.5 56.2
KT795361 53.9 60.0 57.9 46.7 60.0 64.3 52.5 56.0
S40599 46.1 0.0 47.4 45.2 0.0 0.0 53.3 48.0
AF313759 52.4 0.0 52.6 51.2 57.9 68.4 53.1 52.4
AF313760 52.4 0.0 52.6 51.2 57.9 68.4 53.1 52.4
AF313762 52.4 0.0 52.6 51.2 57.9 68.4 53.1 52.4
AF313763 47.6 0.0 47.4 51.2 57.9 68.4 53.1 47.6
HM588697 50.0 0.0 52.6 41.9 0.0 0.0 51.1 52.0
MT552112 50.0 0.0 52.6 41.9 0.0 0.0 51.1 52.0
Probe 1
Probe 2
Probe 3
Probe 4
Probe 5
Probe 6
Probe 7
Probe 8
Probes
Percentage Identity (%)
0
10
20
30
40
50
60
